# Not Your Usual Culprit: Relapsing Salmonella enterica Serovar Infantis Urinary Tract Infection

**DOI:** 10.7759/cureus.93577

**Published:** 2025-09-30

**Authors:** Tania Islam, Darshana Wickramasinghe, Usman Ul Haq, KH Imranul Alam

**Affiliations:** 1 Internal Medicine, Medway NHS Foundation Trust, Gillingham, GBR; 2 Microbiology, Medway NHS Foundation Trust, Gillingham, GBR

**Keywords:** antibiotic stewardship, chronic kidney disease, immunocompromised host, recurrent uti, salmonella infantis, stool culture, urinary tract infection, whole genome sequencing, zoonotic exposure

## Abstract

Urinary tract infection due to *Salmonella enterica* serovar Infantis (S. Infantis) is exceptionally rare and typically linked to host vulnerabilities. We report a case of a woman in her 60s with metastatic renal cancer, diabetes, chronic kidney disease, and prior splenectomy who developed three episodes of UTI within a year. Repeated urine cultures grew S. Infantis, and whole-genome sequencing confirmed relapse from intestinal colonization. After sequential short antibiotic courses, sustained resolution followed a seven-day regimen combined with reinforced pet-related hygiene measures. This case highlights the importance of considering unusual uropathogens, using genomic tools to confirm persistence, and addressing potential zoonotic reservoirs.

## Introduction

Non‑typhoidal *Salmonella* (NTS) UTIs account for <0.1% of urinary infections [1,2] and are reported more commonly in older adults or in those with immunosuppression or structural genitourinary disease [1-4]. Genome sequencing has transformed *Salmonella* surveillance by accurately defining relatedness and aiding in outbreak detection and case linkage [5,6]. Beyond its rarity as a uropathogen, serovar Infantis (S. Infantis) has drawn attention for increasing multidrug resistance mediated by plasmid of emerging S. Infantis (pESI)‑like megaplasmids [7]. Companion animals can intermittently shed *Salmonella*, and household exposures are relevant in vulnerable patients [8]. We report recurrent S. Infantis cystitis in an immunocompromised adult, with genome sequencing confirming relapse from intestinal colonization and resolution after culture‑directed therapy plus hygiene measures.

## Case presentation

A woman in her 60s presented on three occasions over approximately one year with dysuria and urgency without systemic features. Past medical history included metastatic renal clear cell carcinoma (Left nephrectomy), splenectomy, thyroidectomy, distal pancreatectomy, type 2 diabetes, chronic kidney disease, hypertension, and gout. There were no recent urological procedures. She reported close contact with a neighbour’s dog that developed gastrointestinal symptoms shortly before her first urinary episode.

At each presentation, she was afebrile and haemodynamically stable. Across all three episodes, midstream urine cultures yielded Salmonella enterica. In Episodes 1 and 3, isolates were serotyped as *Salmonella enterica* serovar Infantis with susceptibility to amoxicillin, ciprofloxacin, and trimethoprim‑sulfamethoxazole. In Episode 2, the urine isolate was reported only as *Salmonella enterica* species/group (not typed), but a concurrent stool culture confirmed intestinal carriage of S. Infantis, and later whole‑genome sequencing showed clonal relatedness across episodes with susceptibility to amoxicillin, ciprofloxacin, and trimethoprim‑sulfamethoxazole. A stool culture confirmed intestinal carriage. Whole‑genome sequencing performed by the reference laboratory showed the isolates were clonally related across episodes, indicating relapse from a persistent reservoir rather than serial reinfections.

Treatment across episodes comprised: initial empiric nitrofurantoin (three days), then ciprofloxacin (five days), and finally co‑trimoxazole (seven days) paired with reinforced hygiene measures; after the final course, she had durable clinical resolution on follow‑up.

Clinical timeline

The patient's first episode involved dysuria and urgency; urine culture grew S. Infantis (susceptible to amoxicillin, ciprofloxacin, trimethoprim‑sulfamethoxazole). A course of three‑day nitrofurantoin gave temporary relief (Table 1).

**Table 1:** Episode 1 – First UTI Episode UTI: urinary tract infection

Sample type	Organism isolated	Serotype / subspecies	WBC (cells/µL)	RBC	Epithelial	Antibiotic susceptibility (S = Sensitive)
Midstream urine	*Salmonella enterica* serovar Infantis	Subspecies I, ST 32, EBG 31	3206	1267	363	Amoxicillin (S), ciprofloxacin (S), piperacillin–tazobactam (S), trimethoprim‑sulfamethoxazole (S)

The second episode involved recurrent cystitis the following month; *Salmonella* was re‑isolated from urine, and the stool culture confirmed intestinal carriage. She was treated with five‑day ciprofloxacin with transient improvement. Post‑treatment urine showed no growth (Table 2).

**Table 2:** Episode 2 – Relapse and Persistent Infection

Sample type	Organism isolated	Serotype / Subspecies	WBC (cells/µL)	RBC	Epithelial	Antibiotic susceptibility (S = Sensitive)	Notes
Midstream urine	Salmonella enterica species	Not typed	2355	1201	640	Consistent with previous	Relapse
Midstream urine	Salmonella enterica group	Not typed	1242	224	13	Ciprofloxacin (S)	Persistent infection
Stool culture	Salmonella enterica serovar Infantis	Subspecies I, ST 32, EBG 31	—	—	—	—	Confirms intestinal colonisation
Midstream urine	No growth	—	6	0	3	—	Post‑treatment clearance

Later that year, the patient experienced her third episode with S. Infantis; genome sequencing showed clonally related isolates across episodes, indicating relapse. Seven‑day co‑trimoxazole with reinforced hygiene led to durable resolution (Table 3).

**Table 3:** Episode 3 – Relapse WGS: whole genome sequencing

Sample type	Organism isolated	Serotype / Subspecies	WBC (cells/µL)	RBC	Epithelial	Antibiotic susceptibility (S = Sensitive)	Notes
Mid-stream urine	Salmonella enterica serovar Infantis	Serotype 32, Subspecies I	7039	53	13	Amoxicillin (S), ciprofloxacin (S), trimethoprim-sulfamethoxazole (S)	Confirmed by WGS as relapse

## Discussion

NTS UTIs are uncommon and should prompt clinicians to look for host vulnerabilities or structural genitourinary abnormalities [1-4]. In this patient, multiple risk factors - diabetes, chronic kidney disease, and splenectomy - likely contributed to susceptibility. Recurrent isolation of the same serovar with identical susceptibility profiles raised suspicion for relapse; genome sequencing confirmation of clonality across episodes provided definitive evidence of persistent colonization rather than serial reinfection [5,6]. This microbiological clarity supported a management strategy integrating source control (hygiene) with targeted therapy. Although S. Infantis is classically enteric, its urinary isolation has been documented in small series and case reports [1-4]. The serovar’s emerging antimicrobial resistance, driven by pESI‑like megaplasmids, underscores the importance of obtaining culture and susceptibility data rather than assuming resistance phenotypes a priori [7]. Companion animals can intermittently shed Salmonella; targeted hygiene counseling is a pragmatic, low‑risk intervention - particularly in immunocompromised hosts [8]. Stewardship principles recommend narrowing therapy to the cultured organism and using the shortest effective duration compatible with clinical improvement. National Institute for Health and Care Excellence (NICE) guidance for lower UTIs and Infectious Diseases Society of America (IDSA) complicated UTI guidance both emphasize culture‑directed therapy and prudent durations, individualized to context [9,10].

Limitations include the absence of imaging (reserved for further recurrences or complication signals) and the lack of microbiological testing of the neighbour’s dog; nonetheless, the temporal association and patient carriage make a domestic reservoir plausible. Additionally, vascular aneurysms and other endovascular foci are recognized as potential reservoirs of Salmonella in invasive infections and should be considered in patients with systemic or invasive features, although our patient remained clinically stable without evidence of such involvement.

## Conclusions

Recurrent* Salmonella enterica *serovar Infantis UTI in an immunocompromised adult most likely reflects relapse from persistent intestinal colonization. Genome sequencing to confirm clonality and stool culture to document carriage can guide source control. Culture‑directed, short‑but‑effective therapy paired with rigorous hygiene achieved durable resolution; clinicians should elicit animal exposures and counsel on practical hygiene. Future studies should investigate the possible zoonotic origin of *Salmonella* Infantis in recurrent urinary tract infections.
